# Characterization of *Acinetobacter baumannii-calcoaceticus* complex isolates and microbiological outcome for patients treated with sulbactam-durlobactam in a phase 3 trial (ATTACK)

**DOI:** 10.1128/aac.01698-23

**Published:** 2024-04-03

**Authors:** Alita A. Miller, Samir H. Moussa, Sarah M. McLeod

**Affiliations:** 1 Entasis Therapeutics Inc., an affiliate of Innoviva Specialty Therapeutics, Inc., Waltham, Massachusetts, USA; Johns Hopkins University School of Medicine, Baltimore, Maryland, USA

**Keywords:** sulbactam-durlobactam, *Acinetobacter baumannii*, β-lactamase inhibitor

## Abstract

*Acinetobacter baumannii-calcoaceticu*s complex (ABC) causes severe, difficult-to-treat infections that are frequently antibiotic resistant. Sulbactam-durlobactam (SUL-DUR) is a targeted β-lactam/β-lactamase inhibitor combination antibiotic designed to treat ABC infections, including those caused by multidrug-resistant strains. In a global, pathogen-specific, randomized, controlled phase 3 trial (ATTACK), the efficacy and safety of SUL-DUR were compared to colistin, both dosed with imipenem-cilastatin as background therapy, in patients with serious infections caused by carbapenem-resistant ABC. Results from ATTACK showed that SUL-DUR met the criteria for non-inferiority to colistin for the primary efficacy endpoint of 28-day all-cause mortality with improved clinical and microbiological outcomes compared to colistin. This report describes the characterization of the baseline ABC isolates from patients enrolled in ATTACK, including an analysis of the correlation of microbiological outcomes with SUL-DUR MIC values and the molecular drivers of SUL-DUR resistance.

## INTRODUCTION

*Acinetobacter baumannii* is a member of the *Acinetobacter baumannii-calcoaceticus* complex (ABC), a group of closely related *Acinetobacter* species that can cause serious infections and are frequently multidrug resistant (MDR) (1). Carbapenem-resistant *A. baumannii* is currently the fifth leading cause of death attributable to antimicrobial resistance around the world (2) and has been identified as a priority pathogen for the development of new antibiotics (3). Sulbactam-durlobactam (SUL-DUR) is a β-lactam/β-lactamase inhibitor combination antibiotic with targeted activity against ABC (4). Although sulbactam is most commonly used as a β-lactamase inhibitor in combination with a β-lactam (such as ampicillin-sulbactam), it has unique antibacterial activity against ABC due to inhibition of penicillin-binding proteins (5). Unfortunately, the clinical utility of sulbactam for the treatment of ABC is limited due to its susceptibility to a number of β-lactamases commonly expressed in this pathogen which render it inactive (6). Durlobactam is a broad-spectrum diazabicyclooctane inhibitor of Ambler class A, C, and D serine β-lactamases that can restore the activity of sulbactam against contemporary carbapenem-resistant ABC (CRABC) (4). In multiple surveillance studies, the MIC_90_ of SUL-DUR against ABC ranged from 2 to 4 µg/mL (7–10).

In a global phase 3 trial (ATTACK), SUL-DUR met the criteria for non-inferiority versus colistin for 28-day all-cause mortality in patients with hospital-acquired bacterial pneumonia or ventilator-associated bacterial pneumonia, ventilated pneumonia, or bloodstream infections due to CRABC (11). ATTACK was conducted in two parts: Part A was an assessor-blind, randomized, comparative trial that evaluated the efficacy and safety of SUL-DUR compared with colistin in patients with documented ABC. Part B was an open-label, observational study for patients with ABC infections who did not tolerate colistin or whose ABC pathogens were resistant to colistin and who were treated with SUL-DUR. The following report summarizes the characterization of the baseline ABC isolates from patients enrolled in the ATTACK trial, including an analysis of the correlation of microbiological outcome with SUL-DUR MIC and the molecular drivers of SUL-DUR resistance.

## RESULTS

### ABC baseline isolates in ATTACK were primarily *A. baumannii* and highly antibiotic resistant

**Fig 1:**
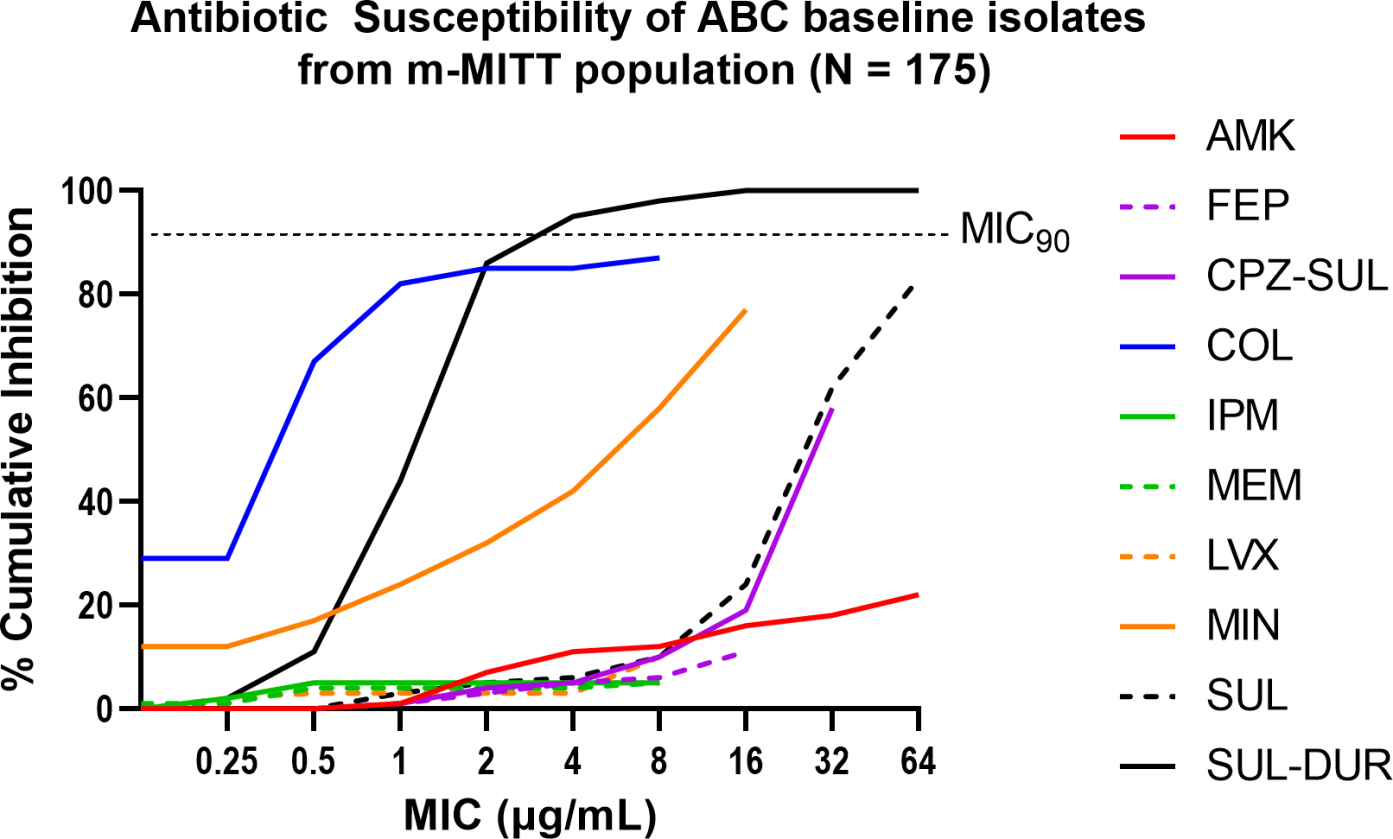
Cumulative percent inhibition of 175 ABC baseline isolates by MIC of sulbactam-durlobactam and comparator antibiotics. AMK = amikacin, FEP = cefepime, CPZ-SUL = cefoperazone-sulbactam (2:1), COL = colistin, IPM = imipenem, LVX = levofloxacin, MEM = meropenem, MIN = minocycline, SUL = sulbactam, and SUL-DUR = sulbactam-durlobactam. Sulbactam-durlobactam was tested as a titration of sulbactam in the presence of 4 µg/mL durlobactam.

A total of 183 Part A and B patients from 59 clinical sites in 16 countries were enrolled in the microbiologically modified intent-to-treat (m-MITT) population (those who received any amount of study drug and had an ABC organism isolated at baseline) (11). Species identification by central and local laboratories showed that 143 (78% of 183) of ABC baseline isolates from m-MITT patients were identified as *Acinetobacter baumannii*, four (2.2%) were *A. dijkshoorniae*, and three (1.6%) were *A. nosocomialis*. The remaining ~20% of isolates were speciated only as ABC. One hundred seventy-five of these 183 isolates were sent to the central laboratory for comprehensive antibiotic susceptibility testing and were found to be highly antibiotic resistant, based on CLSI interpretive criteria (12). Ninety-six percent of these isolates were non-susceptible to carbapenems and ≥85% were non-susceptible to all other agents tested except for minocycline and colistin, for which 43% were non-susceptible to minocycline and 17% were resistant to colistin (MIC ≥ 4 µg/mL) (Table 1; Fig. 1). Based on proposed international definitions (13), 168 (96%) were MDR, 148 (85%) were extensively drug resistant (XDR), and 26 (15%) were pan drug resistant (PDR) (Table 2). Although 167 (95.4%) of ABC baseline isolates from 175 m-MITT patients were non-susceptible to sulbactam alone (MIC > 4 µg/mL), only 8 (4.6%) were non-susceptible to SUL-DUR (MIC > 4 µg/mL) (Table 1; Table S2), including just one of the 26 PDR isolates. Therefore, durlobactam restored the activity of sulbactam against 95.2% (159 of 167) of sulbactam-non-susceptible ABC isolates from ATTACK.

**TABLE 1:** Antibiotic susceptibilities of 175 ABC baseline isolates from m-MITT patients in ATTACK^*a*^

Antibacterial agent	MIC (µg/mL)			% NS (CLSI)
	Range	MIC_50_	MIC_90_	
Amikacin	1 to >64	>64	>64	85
Cefepime	1 to >16	>16	>16	95
Cefoperazone-sulbactam, 2:1	1 to >32	32	>32	NA
Colistin	≤0.25 to >8	0.5	>8	17^*b*^
Imipenem	0.12 to >8	>8	>8	96
Meropenem	0.06 to >8	>8	>8	96
Levofloxacin	0.06 to >4	>4	>4	96
Minocycline	≤0.12 to >16	4	16	43
Tigecycline	0.06 to >4	1	2	NA
Sulbactam	1 to >64	32	>64	NA
Sulbactam-durlobactam	0.25–16	2	4	4.6

^*a*^ ABC = *Acinetobacter baumannii-calcoaceticus* complex, which includes 175 out of the 183 isolates that were available for susceptibility testing at the Central Lab; CLSI = Clinical and Laboratory Standards Institute; NA = not applicable because CLSI breakpoints are not available; and NS = non-susceptible according to CLSI breakpoint criteria (12).

^*b*^ As no CLSI susceptible interpretive criteria exist for colistin (only intermediate and resistant), percent resistance is reported (12). Sulbactam-durlobactam was tested as a titration of sulbactam in the presence of 4 µg/mL durlobactam.

**TABLE 2:** *In vitro* activity of sulbactam-durlobactam against resistant subsets of ABC baseline isolates from ATTACK^*a*^

Category	ABC baseline isolates, *N* (%)	SUL-DUR MIC range (µg/mL)	SUL-DUR MIC_50/90_ (µg/mL)
ALL	175 (100)	0.25–16	2/4
CARB-R	168 (96)	0.5–16	2/4
MDR	168 (96)	0.5–16	2/4
XDR	148 (85)	0.5–16	2/4
PDR	26 (15)	1–8	2/4

^*a*^ CARB‑R = carbapenem-resistant (MIC > 4 µg/mL); MDR = non-susceptible to at least three different antibiotic classes used to treat ABC; PDR = non-susceptible to all tested antibiotic classes approved for use to treat ABC; XDR = non‑susceptible to all but two antibiotic classes approved for use to treat ABC (13). Sulbactam-durlobactam was tested as a titration of sulbactam in the presence of 4 µg/mL durlobactam.

These patterns of susceptibility to SUL-DUR and comparators were consistent across geographic regions, except that of colistin, which was variable (Table S1). Colistin non-susceptibility of ABC isolates ranged from 30% in Europe to 0% in Latin America and China. ABC isolated from bloodstream infections had notably higher colistin and minocycline non-susceptibility rates (57% and 62%, respectively) compared to respiratory isolates (10% and 40%, respectively). Slightly higher rates of SUL-DUR non-susceptibility were observed in ABC isolates from China (12%).

### Minimal effect of imipenem on sulbactam-durlobactam activity against ABC isolates from ATTACK

**Fig 2:**
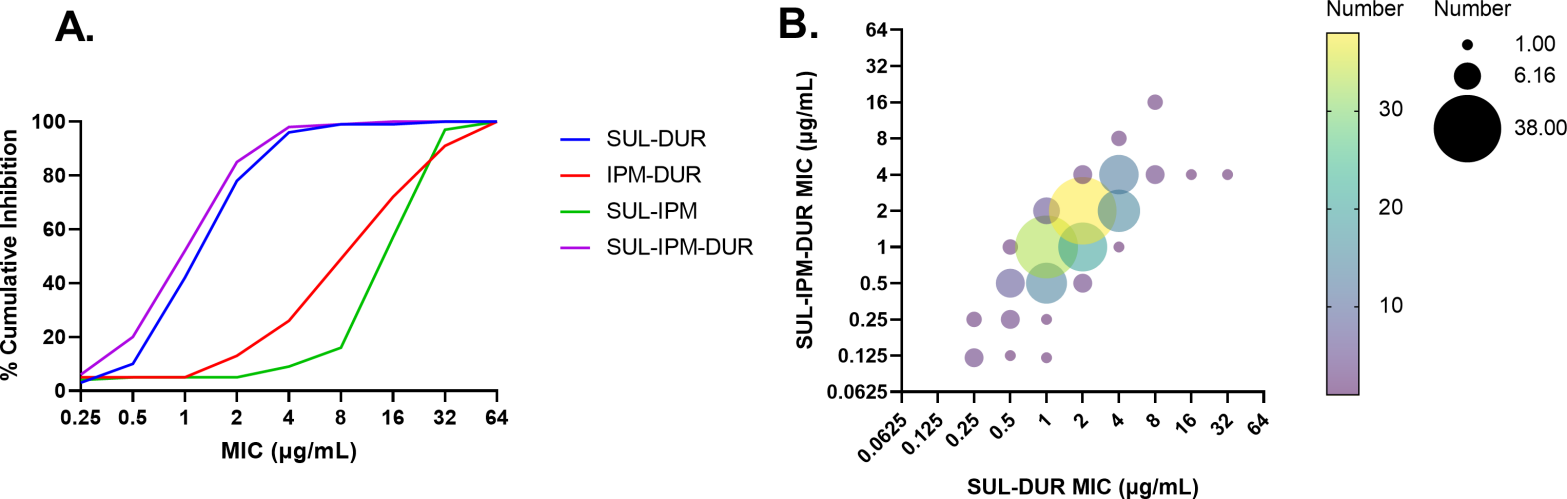
Comparison of sulbactam-durlobactam versus imipenem-sulbactam-durlobactam MIC values for 175 baseline ABC isolates from ATTACK. (A) Cumulative percent inhibition by MIC. (B) Bubble graph comparing MIC values of SUL-IPM-DUR to SUL-DUR. MIC values were determined as follows: SUL-DUR = titration of sulbactam in the presence of 4 µg/mL durlobactam; IPM-DUR = titration of imipenem in the presence of 4 µg/mL durlobactam; SUL-IPM = titration of a 1:1 ratio of sulbactam:imipenem; SUL-IPM-DUR = titration of a 1:1 ratio of sulbactam:imipenem in the presence of 4 µg/mL durlobactam.

Because the activity of SUL-DUR is targeted to ABC, imipenem (IPM)-cilastatin was included as background therapy in ATTACK to treat any co-infecting non-ABC organisms that may be present in polymicrobial infections (11). Since durlobactam inhibits Ambler class A and D carbapenemases such as KPC and OXA families of enzymes (4), it was considered possible that durlobactam might enhance the activity of imipenem (in addition to sulbactam) against ABC infecting organisms when all three agents were dosed together. Accordingly, the activity of IPM, SUL, and DUR alone or in double and triple combinations was tested by broth microdilution against the 175 ABC isolates described above to address the contribution of each component to the observed antibacterial activity *in vitro*. As shown in Table 3, all three agents were inactive against this collection when tested alone (MIC_90_ values ≥ 64 µg/mL). The MIC_50/90_ values for both IPM-SUL and IPM-DUR were 16/32 µg/mL, eightfold higher than that observed for SUL-DUR (MIC_50/90_ = 2/4 µg/mL). The MIC_90_ value for IPM-SUL-DUR was 4 µg/mL, identical to the MIC_90_ value of SUL-DUR alone, while the MIC_50_ value of this triple combination decreased by one doubling dilution compared to SUL-DUR, to 1 µg/mL (Table 3). To characterize the effect of adding IPM to SUL-DUR on antibacterial activity against the ABC isolates from ATTACK more precisely, the MIC values of SUL-DUR with and without IPM were compared in two different ways: by cumulative percent inhibition and in a bubble graph (Fig. 2). Both of these comparisons demonstrate that the overall *in vitro* activity of SUL-DUR against this set of 175 baseline ABC isolates is nearly identical with or without IPM added. In the bubble graph (Fig. 2B), most isolates either had the same MIC value for SUL-DUR and IPM-SUL-DUR combinations or showed a twofold increase or decrease in MIC values (which is within the error of the assay). Addition of imipenem decreased the SUL-DUR MIC value for five of the eight SUL-DUR-non-susceptible isolates by twofold or more (Table S2). Importantly, no antagonism was seen with the addition of IPM to SUL-DUR.

**TABLE 3:** *In vitro* susceptibility of 175 ABC baseline isolates in ATTACK to sulbactam, durlobactam, and imipenem alone or in combination^*a*^

Antibacterial agent or combination	MIC range (µg/mL)	MIC_50_ (µg/mL)	MIC_90_ (µg/mL)
Sulbactam	0.25 to >64	32	64
Durlobactam	0.25 to >64	64	>64
Sulbactam-durlobactam	0.25–32	2	4
Imipenem	0.12 to >64	64	>64
Imipenem-durlobactam	0.12–64	16	32
Sulbactam-imipenem (1:1)	0.12–64	16	32
Sulbactam-imipenem (1:1) + durlobactam	0.12–16	1	4

^*a*^ Durlobactam was held at a fixed concentration of 4 µg/mL in all combinations tested.

### Microbiological outcomes by SUL-DUR MIC for patients treated with SUL-DUR

The SUL-DUR MIC value of the baseline ABC isolate from each evaluable patient (patients in the CRABC m-MITT with SUL-DUR MIC < 8 µg/mL) treated with SUL-DUR in ATTACK was compared to the patient’s microbiological outcome at test of cure (TOC; 7 ± 2 days after last dose). A microbiological response was defined as microbiological eradication or presumed eradication (patient experienced clinical cure and there was no microbiological sample taken) (11). Baseline ABC isolates from 61 of the 63 patients in the Part A SUL-DUR arm were available for SUL-DUR susceptibility testing by the central laboratory and were included as part of the evaluable patient population (Table 4). In Part B, ABC baseline isolates from 26 of 28 patients were susceptible to SUL-DUR (MIC ≤ 4 µg/mL) (Table 4). Across patients in Parts A and B, there were five whose baseline isolates tested with a SUL-DUR MIC of 0.5 µg/mL, 28 with an MIC of 1 µg/mL, 43 with an MIC of 2 µg/mL, and 11 with an MIC of 4 µg/mL (Table 4). For patients treated with SUL-DUR, the overall eradication or presumed eradication rate at TOC for patients with SUL-DUR-susceptible ABC isolates (MIC ≤ 4 µg/mL) was 72% (63 of 87 patients). Similar microbiological response rates were observed across MIC categories: 60% at 0.5 µg/mL, 68% at 1 µg/mL, 75% at 2 µg/mL, and 82% at 4 µg/mL.

**TABLE 4:** Microbiological outcomes of evaluable patients treated with SUL-DUR by SUL-DUR MIC of ABC baseline isolates in ATTACK

		SUL-DUR MIC of baseline ABC (µg/mL)			
	Total, N (%)	0.5	1	2	4
All evaluable patients who received SUL-DUR**^*a*^**					
Number of patients	87	5	28	43	11
(Presumed) Eradication	63 (72%)	3 (60%)	19 (68%)	32 (75%)	9 (82%)
(Presumed) Persistence	18 (21%)	2 (40%)	5 (18%)	10 (23%)	1 (9%)
Indeterminate	6 (7%)	0	4 (14%)	1 (2%)	1 (9%)
Part A CRABC m-MITT					
Number of patients**^*b*^**	61	4	22	28	7
(Presumed) Eradication	42 (69%)	2 (50%)	14 (64%)	20 (71%)	6 (86%)
(Presumed) Persistence	15 (24%)	2 (50%)	5 (23%)	8 (28%)	0
Indeterminate	4 (6%)	0	3 (14%)	0	1 (14%)
Part B					
Number of patients	26	1	6	15	4
(Presumed) Eradication	21 (81%)	1 (100%)	5 (83%)	12 (80%)	3 (75%)
(Presumed) Persistence	3 (12%)	0	0	2 (13%)	1 (25%)
Indeterminate	2 (7%)	0	1 (7%)	1 (7%)	0

^*a*^ Patients in the CRABC m-MITT with SUL-DUR MIC < 8 µg/mL.

^*b*^ The SUL-DUR arm in Part A CRABC m-MITT consisted of 61 patients for this analysis because the SUL-DUR MIC was not determined for two baseline ABC isolates that were not sent to the central laboratory. Presumed eradication = patient experienced clinical cure and there was no microbiological sample taken. Presumed persistence = patient experienced clinical failure and there was no microbiological sample taken. Sulbactam-durlobactam was tested as a titration of sulbactam in the presence of 4 µg/mL durlobactam.

Four patients treated with SUL-DUR (two in Part A and two in Part B) had baseline ABC isolates with SUL-DUR MIC values of 8–16 µg/mL (the other four were treated with colistin) (Table S2). Although these patients did not meet inclusion criteria for the CRABC m-MITT population (because of the elevated SUL-DUR MIC values), all four survived to 28 days, three (75%) experienced clinical cure, and two (50%) experienced microbiological eradication at TOC. Similar rates of microbiological responses were observed for patients with XDR and PDR ABC infections treated with SUL-DUR (Table S3).

### ABC isolates with pre-existing SUL-DUR non-susceptibility encode PBP3 variants

To characterize the molecular drivers of SUL-DUR resistance in ABC isolates from patients enrolled in ATTACK, the eight baseline isolates with elevated SUL-DUR MIC values (SUL-DUR MIC > 4 µg/mL) were subjected to whole genome sequencing analysis (Table S2). Four of these isolates originated from four patients treated with SUL-DUR (two in Part A and two in Part B) and the other four isolates originated from patients treated with colistin. The dominant sequence type (ST) as determined using the Institut Pasteur (IP) method was ST_IP_2 (six of eight isolates). Additionally, all strains encoded for an *Acinetobacter*-derived cephalosporinase Ambler class C β-lactamase gene, as well as two different Ambler class D OXA β-lactamase genes (OXA-23 and OXA-51-like carbapenemases), and six of eight isolates encoded an Ambler class A TEM-1 β-lactamase gene (Table S2). All eight isolates with elevated SUL-DUR MIC values encoded for variants with mutations at or near the active site of PBP3, the primary target of sulbactam activity in *A. baumannii* (5): three encoded PBP3 [T526S], three encoded PBP3 [A515V], one encoded PBP3 [G523V], and one encoded PBP3 [N377Y, T526S] (Table S2). In addition, several isolates had mutations in efflux genes or other genes previously associated with resistance to sulbactam or SUL-DUR (14). However, similar efflux mutations have also been observed in SUL-DUR-susceptible isolates (data not shown), so their relative contribution to elevated SUL-DUR MIC values cannot be established using genomic sequencing information alone.

### Emergence of resistance to SUL-DUR was rare in ATTACK

Of the 106 m-MITT patients (78 patients in Part A and 28 patients in Part B) who were treated with SUL-DUR in ATTACK, one (0.9%) had a SUL-DUR susceptible ABC isolate at baseline (MIC = 4 µg/mL) that became non-susceptible over time (Table S4). This infection, which was caused by a PDR ABC isolate, was persistent at TOC, but was found to be eradicated at the late follow-up visit (7 ± 2 days after TOC). In addition, the patient demonstrated clinical cure at TOC and survived to day 28. Addition of imipenem had no effect on the SUL-DUR MIC values of either the baseline or longitudinal ABC isolates from this patient (Table S4). The baseline ABC isolate encoded the A515V variant of PBP3 (Table S4). Because SUL-DUR-susceptible ABC isolates that encode the PBP3 A515V variant have been observed and biochemical results suggest the PBP3 A515V variant retains 55% sulbactam-binding activity compared to wild-type PBP3 (14), this mutation alone appears to be insufficient to confer SUL-DUR resistance, although it is likely to be a contributing factor, depending on other molecular characteristics of the isolate. The only discernable genetic difference in the longitudinal isolates compared to the baseline isolate from this patient was a G288S mutation in AdeJ. This variant was recently described as being associated with SUL-DUR non-susceptibility in both an *A. baumannii* isolate from a global surveillance study (14) and a compassionate use case report where the patient was infected with an ABC isolate that had acquired this AdeJ mutation prior to receiving SUL-DUR therapy (15). Because durlobactam has been shown to be a substrate of the AdeIJK efflux system (14, 16), it is likely that the molecular driver of SUL-DUR resistance in this isolate was due to both the PBP3 A515V variant and a decrease in the effective intracellular concentration of durlobactam due to AdeJ-mediated efflux.

## DISCUSSION

A notable characteristic of the ABC isolates from patients enrolled in ATTACK was the high rate of antibiotic resistance compared to results described in recent surveillance studies. ABC isolates from a 6-year global surveillance study (10) were significantly more susceptible to comparator antibiotics than those isolated from patients enrolled in the ATTACK phase 3 trial. Of particular interest was the difference in carbapenem susceptibility: approximately 50% of ABC isolates from the global surveillance study were carbapenem-non-susceptible whereas 96% of ABC isolates from m-MITT patients in ATTACK were carbapenem-non-susceptible. Of note, in the surveillance study, carbapenem susceptibility varied from approximately 30% to 72% resistant, depending on the region, which could help explain the high rate of carbapenem resistance among ATTACK patients as the trial was conducted primarily in regions with higher rates of antibiotic resistance. Colistin non-susceptibility was also much higher in ABC isolates from ATTACK (17%) as compared to those in the global surveillance study (4.1%) (10). In contrast to most of the comparator antibiotics, SUL-DUR was equally active against the ABC isolates from both studies, with ≥95% of ABC isolates in each demonstrating sulbactam-durlobactam MIC values ≤4 µg/mL (17). A comparison of MDR, XDR, and PDR rates between ABC isolates from the global surveillance study and isolates from ATTACK is shown in Table S5. SUL-DUR retained potent activity (MIC_90_ value of 4 µg/mL) against all MDR, XDR, and PDR subsets of both collections of ABC isolates (Table S5).

Although durlobactam is a potent inhibitor of Ambler class A, C, and D serine β-lactamases, it does not inhibit class B metallo-β-lactamases (MBLs) (4). It was therefore reassuring to observe that none of the SUL-DUR-non-susceptible isolates in ATTACK encoded MBL genes. This low incidence is consistent with the rate of MBL-encoding ABC in the 6-year global surveillance studies, in which only 0.7% (33 of 5,032) of isolates were SUL-DUR-non-susceptible due to MBL expression (14). However, it is important to note that MBL-encoding ABC outbreaks have been reported in certain geographic regions such as India and are a growing concern in Latin America (18, 19); therefore, close monitoring for the potential international spread of these clones is imperative.

Of the 175 ABC baseline isolates from the ATTACK trial that were tested for susceptibility to sulbactam-durlobactam, only eight isolates were found to be non-susceptible to sulbactam-durlobactam (MIC values of 8–16 µg/mL). All eight isolates with elevated SUL-DUR MIC values encoded for variants with mutations at or near the active site of PBP3, the primary target of sulbactam activity in *A. baumannii* (5). Of note, in the presence of imipenem, the SUL-DUR MIC value for seven out of eight of these isolates was reduced to ≤4 µg/mL, the sulbactam-durlobactam susceptible breakpoint. Similar PBP3 variants have been observed in surveillance studies (14, 20–22). In a global 6-year study, isolates with PBP3 variants that were associated with elevated sulbactam-durlobactam MIC values comprised ~1% of isolates tested (10, 14). In addition to PBP3 variants identified in baseline isolates, one patient (out of 106 m-MITT patients in Part A and Part B treated with SUL-DUR) developed resistance during SUL-DUR treatment in the ATTACK study. Longitudinal isolates collected from this patient had acquired a single missense mutation in the AdeJ efflux pump at amino acid position G288S. Alteration of AdeJ at this position has been shown previously to decrease susceptibility to SUL-DUR, likely due to increased efflux potential (14, 15). Therefore, PBP3 mutations that affect sulbactam binding to its target and efflux-related mutations that lead to elevated SUL-DUR MICs should both be evaluated for SUL-DUR non-susceptible ABC isolates in future surveillance studies.

The high rates of microbiological responses at TOC for patients who received SUL-DUR therapy are consistent with improved clinical outcomes observed in ATTACK as previously described (11). High rates of positive microbiological outcomes were observed for all SUL-DUR MIC categories from 0.5 to 4 µg/mL. Microbiological eradication rates did not correlate to the SUL-DUR MIC for MIC values of ≤4 µg/mL, i.e., higher rates of eradication were observed in patients infected with ABC isolates with a SUL-DUR MIC of 4 µg/mL than was seen with patients whose ABC isolates had a SUL-DUR MIC of 0.5 µg/mL. This is likely due to the relatively small patient numbers at the lower MICs. Of note, favorable clinical and microbiological outcomes were observed for four patients with SUL-DUR non-susceptible baseline ABC isolates (MIC = 8–16 µg/mL) who received SUL-DUR therapy, which supports the intermediate susceptibility breakpoint for SUL-DUR of 8 µg/mL which was recently FDA approved (17).

The limitations of this study include the relatively small number of patients (in particular when grouping by SUL-DUR MIC of the infecting ABC isolate) and the absence of long-term follow-up beyond 28 days of the study. While ATTACK was a global study, it is possible that different clones of ABC from underrepresented parts of the globe could influence outcomes.

In summary, the microbiological data presented here provide further support for the use of SUL-DUR for the treatment of hospital-acquired and ventilator-associated pneumonia caused by carbapenem-resistant ABC.

## MATERIALS AND METHODS

### Species identification and antibiotic susceptibility testing

The central laboratory (International Health Management Associates, Inc., Schaumburg, IL, USA) received 175 of 183 isolates. A small subset of clinical sites was unable to ship isolates due to the coronavirus disease-19 pandemic. In these cases, species determination and carbapenem susceptibility data generated at the local laboratory were used to determine a patient’s eligibility for enrollment. Isolates received by the central laboratory were sub-cultured, and speciation was performed by matrix-assisted laser desorption ionization–time of flight (MALDI-TOF) mass spectrometry. If the culture was mixed or yielded no growth, a back-up sample was requested. Minimum inhibitory concentration endpoints were determined by broth microdilution following CLSI guidelines (23). The testing paradigm for the combinations tested was as follows: for SUL-DUR, sulbactam was titrated in the presence of 4 µg/mL durlobactam; for IPM-DUR, imipenem was titrated in the presence of 4 µg/mL durlobactam; SUL-IPM, titration of a 1:1 ratio of sulbactam:imipenem; SUL-IPM-DUR, titration of a 1:1 ratio of sulbactam:imipenem in the presence of 4 µg/mL durlobactam; CPZ-SUL, titration of a 2:1 ratio of cefoperazone:sulbactam. Quality control of broth microdilution panels followed CLSI guidelines and used ranges approved for SUL-DUR and all comparative agents using the following strains: *Escherichia coli* ATCC 25922, *Pseudomonas aeruginosa* ATCC 27853, and *A. baumannii* NCTC 13304 (12). Results were interpreted using CLSI susceptibility criteria (12). Although CLSI does not recognize a susceptible breakpoint for colistin (12), ABC isolates with colistin MIC values of <4 µg/mL were classified as susceptible for the purposes of this clinical trial.

### Whole genome sequencing and analysis

All ABC isolates with SUL-DUR MIC results of >4 mg/L (with durlobactam fixed at 4 µg/mL) were subjected to whole genome sequencing. ABC clinical trial isolates from the same species collected throughout a patient’s treatment course [screening and TOC/late follow-up (LFU) visits] were analyzed for genetic relatedness [multilocus sequence typing (MLST)] to differentiate between re-infection and persistent infection. Extraction of chromosomal DNA and whole genome sequencing were performed by JMI Laboratories (North Liberty, IA, USA), or in the case of isolates from China, sequencing was performed by BGI Genomics (Shenzhen, China). Subsequent analysis of genomic data was performed at both JMI laboratories and Entasis Therapeutics Inc. Chromosomal DNA was extracted from each isolate using the Thermo Scientific KingFisher Flex Magnetic Particle Processors (Cleveland, OH, USA) following the manufacturer’s instructions. Total genomic DNA libraries were prepared for sequencing using the Nextera sample preparation kit and Nextera index primers, following the manufacturer’s instructions (Illumina, San Diego, CA, USA) (Promega, Madison, WI, USA). Samples were sequenced on an Illumina MiSeq instrument using V2 or V3 chemistry.

Assembly and analysis of whole genome sequencing were performed using CLC Genomics Workbench v22.0 (Qiagen). Fastq files were processed and analyzed as follows: duplicate sequence reads were removed, and the remaining reads were trimmed for quality and minimum length (50 bp). Reads were *de novo* assembled at high stringency (fraction length = 0.9, similarity fraction = 0.99) using default mismatch/insertion/deletion costs.

Strains with reduced susceptibility to SUL-DUR were selected for sequence analysis of cell wall synthesis, efflux, porin, as well as other genes of interest. The corresponding amino acid sequences were compared with the reference sequence of *A. baumannii* strain ATCC 17978 (Genbank accession number CP000521.1). The resultant variations in the amino acids of the proteins are listed in Tables S2 and S4. The β-lactamase content of each strain was determined by BLAST within the CLC Genomics Workbench against an assembled database of genes curated at Entasis Therapeutics Inc. with sequences originating from the NCBI Bacterial Antimicrobial Resistance Reference Gene Database (accession number PRJNA313047). For MLST determination, assembled contigs were exported from CLC Genomics Workbench and uploaded into the PubMLST database (https://pubmlst.org/databases/). For *Acinetobacter*, PubMLST hosts two different MLST schemes: Oxford and Institute Pasteur. The Oxford scheme (ST_ox_) assigns sequence types using the following genes: *gltA, gyrB, gdhB, recA, cpn60, gpi,* and *rpoD*. Alternatively, the Institut Pasteur scheme (ST_IP_) assigns sequence types using alleles of *cpn60, fusA, gltA, pyrG, recA, rplB,* and *rpoB*. Sequence types from both schemes are reported when available.

## Data Availability

All genome sequencing data described in this manuscript have been deposited at DDBJ/ENA/Genbank as part of Bioproject PRJNA1049754, under the accession JAXMQG000000000-JAXMQS000000000. The versions described in this paper are versions JAXMQG010000000-JAXMQS010000000.

## SUPPLEMENTAL MATERIAL

Supplemental tables aac.01698-23-s0001.pdf Tables S1 to S5.

ASM does not own the copyrights to Supplemental Material that may be linked to, or accessed through, an article. The authors have granted ASM a non-exclusive, world-wide license to publish the Supplemental Material files. Please contact the corresponding author directly for reuse.
