# Supplementary material for: Characterization of Acinetobacter baumannii-calcoaceticus complex isolates and microbiological outcome for patients treated with sulbactam-durlobactam in a phase 3 trial (ATTACK)
Source: Antimicrob Agents Chemother. 2024 Apr 3;68(5):e01698-23. doi: 10.1128/aac.01698-23 (PMC11064521; doi:10.1128/aac.01698-23)
Supplement: Supplemental tables — Tables S1 to S5. [file aac.01698-23-s0001.pdf]

Characterization of *Acinetobacter baumannii-calcoaceticus* complex isolates and microbiological outcome for patients treated with sulbactam-durlobactam in a phase 3 trial (ATTACK)

Alita A. Miller[a,b], Samir H. Moussa[a] and Sarah M. McLeod[a, #]

[a]Entasis Therapeutics Inc., an affiliate of Innoviva Specialty Therapeutics, Inc.

[b]Author's present affiliation: Arrepath, Inc., 303A College Rd East, Princeton, NJ 08540

[#]To whom correspondence should be addressed:

Sarah M. McLeod

Innoviva Specialty Therapeutics, Inc., an affiliate of Entasis Therapeutics Inc.

35 Gatehouse Drive

Waltham, MA, USA

Sarah.McLeod@istx.com

Supplemental Table 1. Antibiotic susceptibilities of 175 ABC baseline isolates from the m-MITT patient population by region and infection type

Antibiotic	Region (N)	All (N = 175)	Europe (N = 91)	Latin America (N = 16)	Asia-Pacific (N = 25)	China (N = 42)	USA (N = 1)	Respiratory Infection (N = 154)	Bloodstream Infection (N = 21)
Amikacin	MIC Range (µg/mL)	1 - > 64	1 - > 64	2 - > 64	2 - > 64	2 - > 64	>64	1 - > 64	2 - > 64
	MIC_{90} (µg/mL)	>64	>64	NA	>64	>64	NA	>64	>64
	% NS	85	92	87	76	88	NA	87	95
Colistin	MIC Range (µg/mL)	≤0.25 ->8	≤0.25 ->8	≤0.25 - 0.5	≤0.25 - >8	≤0.25 - 2	0.5	≤0.25 - >8	≤0.25 - >8
	MIC_{90} (µg/mL)	>8	>8	NA	2	0.5	NA	8	>8
	% NS	17	30	0	4	0	NA	10	57
Imipenem	MIC Range (µg/mL)	0.12 - > 8	0.12 - > 8	0.12 - > 8	0.5 - > 8	0.12 - > 8	>8	0.12 - > 8	0.25 - > 8
	MIC_{90} (µg/mL)	>8	>8	NA	>8	>8	NA	>8	>8
	% NS	96	94	94	96	95	NA	95	96
Minocycline	MIC Range (µg/mL)	≤0.12 - >16	≤0.12 - >16	≤0.12 - >16	≤0.12 - >16	≤0.12 - >16	16	≤0.12 - >16	≤0.12 - >16
	MIC_{90} (µg/mL)	16	16	NA	8	8	NA	16	16
	% NS	46	49	56	4	28	NA	40	62
Sulbactam	MIC Range (µg/mL)	1 - >64	1 - >64	2 - 64	2 - >64	2 - 64	16	1 - >64	1 - >64
	MIC_{90} (µg/mL)	>64	64	NA	64	>64	NA	>64	>64
	% NS	94	93	94	96	95	NA	94	95
Sulbactam-durlobactam	MIC Range (µg/mL)	0.25 - 16	0.25 - 8	0.5 - 2	0.5 - 8	0.25 - 16	1	0.25 - 16	0.5 - 8
	MIC_{90} (µg/mL)	4	4	NA	2	8	NA	4	4
	% NS	4.6	2	0	4	12	NA	5	5

NA = not applicable. MIC_{90} was not calculated for subsets of N < 20; NS = non-susceptible based on CLSI 2021 breakpoint criteria (CLSI M100-S31, 2021). Regions (countries) in this study were defined as follows: North America (United States); Europe (Belarus, Greece, Hungary, Israel, Lithuania, Russia, and Turkey); Latin America (Brazil, Mexico, and Peru) and Asia Pacific (India, South Korea, Taiwan, and Thailand). China was considered its own region. Sulbactam-durlobactam was tested as a titration of sulbactam in the presence of 4 µg/mL durlobactam.

Supplemental Table 2. Characterization of SUL-DUR-non-susceptible baseline ABC isolates from ATTACK

Country	Patient ID	Isolate ID	Treatment arm	SUL-DUR MIC (µg/mL)	SUL-IPM-DUR MIC (µg/mL)	MLST	PBP3 variant	Encoded β-lactamase genes	Efflux genes	Other Genes
China	SUD-CH-P1	2514067	COL	16*	4	ST_{ox}208 / ST_{IP} 2	T526S	ADC-30; TEM-1; OXA-23; OXA-66	--	--
China	SUD-CH-P2	2514068	SUL-DUR, Part B	8-16*	2	ST_{ox}2499 / ST_{IP}2	T526S	ADC-30; OXA-23; OXA-66	AdeJ [D711N]	--
China	SUD-CH-P3	2427953	COL	8-16*	4	ST_{ox}208 / ST_{IP}2	T526S	ADC-30; TEM-1; OXA-23; OXA-80	--	--
China	SUD-CH-P4	2427959	SUL-DUR	8*	2	ST_{ox}136 / ST_{IP}2	A515V	ADC-73; TEM-1; OXA-23; OXA-66	--	--
China	SUD-CH-P5	2427963	SUL-DUR	8*	2	ST_{ox}195 / ST_{IP}2	A515V	ADC-73; TEM-1; OXA-23; OXA-66	--	--
Taiwan	SUD-TW-P1	2260176	COL	8	4	ST_{ox}789	N377Y, T526S	ADC-115; TEM-1; OXA-23; OXA-66	--	LdtJ [I268N]; RpoC [E502G]
Israel	SUD-IS-P1	2514808	COL	8	4	ST_{ox}106 / ST_{IP}3	G523V	ADC-6-like; OXA-23; OXA-71	AdeJ [A290T]	PBP2 [V132L]; LdtJ [L192H]; VacJ [P47L]
Greece	SUD-GR-P1	2540807	SUL-DUR, Part B	8	8	ST_{ox}425 / ST_{IP}2	A515V	ADC-73; TEM-1; OXA-23; OXA-66	--	--

DUR = durlobactam; MLST = multi-locus sequence type; PBP3 = penicillin binding protein 3; ST_{IP} = sequence type using the Pasteur Institute scheme; ST_{ox} = sequence type using the Oxford scheme; SUL = sulbactam; XDR = extensively drug-resistant; PDR = pan-drug resistant *addition of imipenem decreased SUL-DUR MIC value by >two-fold. SUL-DUR = titration of sulbactam in the presence of 4 µg/mL durlobactam; SUL-IPM-DUR = titration of a 1:1 ratio of sulbactam : imipenem in the presence of 4 µg/mL durlobactam.

Supplemental Table 3. Comparison of SUL-DUR MIC of ABC baseline isolate vs. microbiological outcome for m-MITT patients with XDR or PDR ABC infections treated with SUL-DUR

		SUL-DUR MIC of Baseline ABC (µg/mL)				
	**Overall**	**0.5**	**1**	**2**	**4**	**8**
**m-MITT patients with XDR ABC treated with SUL-DUR**						
**Number of patients**	**67**	**3**	**20**	**32**	**9**	**3**
(Presumed) Eradication	45 (67%)	2 (67%)	13 (65%)	22 (69%)	7 (78%)	1 (33%)
(Presumed) Persistence	18 (27%)	1 (33%)	5 (25%)	9 (28%)	1 (11%)	2 (67%)
Indeterminate	4 (6%)	0	2 (10%)	1 (3%)	1 (11%)	0
**m-MITT patients with PDR ABC treated with SUL-DUR**						
**Number of patients**	**20**	**0**	**1**	**15**	**3**	**1**
(Presumed) Eradication	14 (70%)	0	0	11 (73%)	2 (67%)	1 (100%)
(Presumed) Persistence	5 (25%)	0	0	4 (27%)	1 (33%)	0
Indeterminate	1 (5%)	0	1 (100%)	0	0	0

Presumed eradication = patient experienced clinical cure and there was no microbiological sample taken. Presumed persistence = patient experienced clinical failure and there was no microbiological sample taken. Sulbactam-durlobactam was tested as a titration of sulbactam in the presence of 4 µg/mL durlobactam.

Supplemental Table 4. Characterization of longitudinal ABC isolates from patient SUD-GR-P2 vs. SUL-DUR-susceptible isolates from other patients

Country	Patient ID	Isolate ID	Time of Isolation	MDR, XDR or PDR	SUL-DUR MIC (µg/mL)	SUL-IPM-DUR MIC (µg/mL)	MLST	PBP3 variant	Encoded β-lactamase genes	Efflux genes	Other Genes
	**Single case of emerging resistance to sulbactam-durlobactam in a patient treated with SUL-DUR**										
Greece	SUD-GR-P2	2260380	SCR	PDR	4	4	ST_{ox}436 / ST_{IP}2	A515V	ADC-73 [R172L]; TEM-1; OXA-23; OXA-66		--
		2277699	D5	PDR	8	16	ST_{ox}436 / ST_{IP}2	A515V	ADC-73 [R172L]; TEM-1; OXA-23; OXA-66	AdeJ [G288S]	--
		2277700	D7	PDR	32	16	ST_{ox}436 / ST_{IP}2	A515V	ADC-73 [R172L]; TEM-1; OXA-23; OXA-66	AdeJ [G288S]	--
		2277701	EOT/D8	PDR	16	8	ST_{ox}436 / ST_{IP}2	A515V	ADC-73 [R172L]; TEM-1; OXA-23; OXA-66	AdeJ [G288S]	--
		2277719	TOC/D13	PDR	8	8	ST_{ox}436 / ST_{IP}2	A515V	ADC-73 [R172L]; TEM-1; OXA-23; OXA-66	AdeJ [G288S]	--

D = Day; DUR = durlobactam; EOT = end of treatment; ID = identifier; IPM= imipenem; MIC = minimum inhibitory concentration; MLST = multi-locus sequence type; PBP3 = penicillin binding protein 3; ST_{IP} = sequence type using the Pasteur Institute scheme; SCR = screening (baseline); ST_{ox} = sequence type using the Oxford scheme; SUL = sulbactam; TOC = test of cure; U1 = unscheduled; WT = wild-type. SUL-DUR was tested as a titration of sulbactam in the presence of 4 µg/mL durlobactam; SUL-IPM-DUR was tested as a titration of a 1:1 ratio of sulbactam: imipenem in the presence of 4 µg/mL durlobactam.

Supplemental Table 5. Comparison of MDR, XDR, and PDR rates and sulbactam-durlobactam susceptibility of ABC isolates from a 6-year global surveillance study vs. ATTACK

Study	Category	N (%)	Sulbactam-Durlobactam MIC (µg/mL)			
			Range	MIC_{50}	MIC_{90}	% Susceptible*
Six-Year Global Surveillance[1]	All strains	5,032 (100)	≤0.03 - >64	1	2	98.3
	MDR	2,680 (53.2)	≤0.03 - >64	1	4	96.9
	XDR	2,116 (42.0)	0.25 - >64	2	4	97.2
ATTACK	All strains	175 (100)	0.25 - 16	2	4	95.4
	MDR	168 (96)	0.5 - 16	2	4	95.2
	XDR	148 (85)	0.5 - 16	2	4	94.5
	PDR	26 (15)	1 - 8	2	4	95.6

Abbreviations: ABC = *Acinetobacter baumannii-calcoaceticus* complex; MDR = multidrug resistant; MIC = minimum inhibitory concentration; MIC_{50} = minimum inhibitory concentration required to inhibit the growth of 50% of isolates; MIC_{90} = minimum inhibitory concentration required to inhibit the growth of 90% of isolates; PDR = pan drug resistant; XDR = extensively drug resistant. Sulbactam-durlobactam was tested as a titration of sulbactam in the presence of 4 µg/mL durlobactam.

*based on FDA-approved susceptible breakpoint of ≤4 µg/mL (https://www.fda.gov/drugs/development-resources/sulbactam-and-durlobactam-injection).

[1]Karlowsky JA *et al*. 2022. Antimicrob Agents Chemother. 66(9): e00781-22.
